# Aligning values and behaviours in LGBTQIA+ affirming care: primary care providers’ perspectives

**DOI:** 10.1017/S1463423626101601

**Published:** 2026-08-07

**Authors:** Arin Hectors, Lisa Nissen, Richard Violette, Cylie M. Williams, Alex Ker, Nia Franks, Luke Otto, Corey Wakefield, Megan H. Ross

**Affiliations:** 1 The University of Queensland, Australia; 2 James Cook University, Australia; 3 Monash University, Australia; 4 Victoria University of Wellington, New Zealand

**Keywords:** cultural capability, primary care, sexual and gender minorities, values-driven care

## Abstract

The lesbian, gay, bisexual, transgender, queer, intersex, asexual and other related communities (LGBTQIA+) possess rich and diverse lived experiences yet continue to face systemic barriers to safe and affirming healthcare. This study examined the key features of such care from the perspective of primary care providers who deliver affirming care to LGBTQIA+ people. Thirty-one clinicians across varied disciplines shared their insights through semi-structured interviews. These were analysed using reflexive thematic analysis informed by queer theory and a relativist epistemology. Two overarching concepts were identified across participants’ experiences: their values (purpose, principles and qualities) that underpinned their practices (ways of providing affirming care) that were grounded in intentionality, reflection and relationality. Four key themes were generated that intersected with practices and values: 1) values-driven healthcare, 2) (un)learning biases, reflection, and sociocultural awareness, 3) mitigating the power imbalance in the patient–provider relationship, and 4) valuing lived experience. These themes describe key features of delivering safe and affirming healthcare for participants. The findings highlight the importance of addressing both the observable (behaviours and practices) and unobservable (values) when implementing interventions to improve healthcare provision for LGBTQIA+ people.

## Introduction

The lesbian, gay, bisexual, transgender, queer, intersex, asexual and communities with other related identities and experiences (LGBTQIA+) experience disproportionately higher rates of physical and mental health conditions compared to cisgender, heterosexual and endosex communities. These disparities include elevated risks of depression, anxiety, suicidality, substance use, chronic illness and disability (Mustanski *et al*., 2010; Quinn *et al*., 2015; Edmiston *et al*., 2016). These disparities are not inherent to LGBTQIA+ identities but are shaped by persistent social and structural barriers such as discrimination, stigma and marginalization, including in healthcare settings.

LGBTQIA+ people’s negative healthcare experiences stem from discrimination, in addition to sex, gender and sexuality norms and lack of provider knowledge. This discrimination and knowledge gap contributes to discomfort, feeling unsafe or invalidated, and delaying or avoiding healthcare (Ross and Setchell, 2019). People who are LGBTQIA+ often report having to educate their provider about specific health needs, including sexual health and gender-affirming healthcare (Newman *et al*., 2021). Such encounters not only undermine trust in healthcare provision but perpetuate health disparities (Johnson and Nemeth, 2014; González and Veale, 2024). Conversely, LGBTQIA+ affirming healthcare is characterized by experiences with providers who are knowledgeable, professional, respectful, and inclusive (Smith and Turell, 2017; Ker *et al*., 2022; Withey-Rila *et al*., 2023). Affirming care involves acknowledging a patient’s self-determination and autonomy to define themselves, referring to them as they would like to be referred, and respecting them as a person (Kuzma *et al*., 2019). Affirming healthcare experiences for LGBTQIA+ people can lead to better engagement with healthcare and, in turn, better mental and physical health outcomes (McKay *et al*., 2023).

Provider knowledge, skills and cultural competency are critical determinants of LGBTQIA+ healthcare experiences and outcomes (Pennay *et al*., 2018; Bishop *et al*., 2022; Pickern, 2024). LGBTQIA+ content is often absent or minimally represented within primary care education. As a result, graduates report feeling underprepared and lacking the capabilities to provide safe and affirming care to LGBTQIA+ people (Ross *et al*., 2023; Wynn *et al*., 2024). Many healthcare providers default to ‘equality’ approaches that fail to recognize, and importantly, modify approaches to the contextual and individualized factors that shape LGBTQIA+ experiences and health.

The minority stress model (Meyer, 1995, Meyer, 1995) provides a valuable framework for understanding the importance of adapting approaches to facilitate equity, rather than equality. Although the original minority stress model was developed to explain gay men’s experiences, research has expanded on this to include other LGBTQIA+ communities (Meyer, 2003; Testa *et al*., 2015). The model posits that individuals from marginalized groups experience chronic stress due to societal prejudice, anticipated rejection, internalized stigma, and the need to conceal their identity (Meyer, 1995, Testa *et al*., 2015; Testa *et al*., 2015). These stressors compound over time, contributing to poorer health outcomes and reduced engagement with healthcare services. Thus, it is important for healthcare providers to understand the multiple factors that impact LGBTQIA+ people’s health, and to work towards enacting equitable health practices.

The existing literature confirms that many providers are willing to engage with LGBTQIA+ patients, but lack the training, confidence, and systemic support to successfully provide affirming care (Willging *et al*., 2022; Marshall *et al*., 2023; Jerala and Petek, 2024). In Australia specifically, research indicates that LGBTQIA+ content is minimal and inconsistent across training for various healthcare disciplines including nurses (Huang *et al*., 2024; Priddle *et al*., 2025), occupational therapists (Brown and Ross, 2026), physiotherapists (Ross *et al*., 2023), and in medical schools (Wynn *et al*., 2024). Educational institutions provide a paucity of LGBTQIA+ health content which contributes to healthcare providers in Australia having varied knowledge and approaches to delivering healthcare to LGBTQIA+ people (Ross and Setchell, 2023; Neish and Ross, 2024; Brown and Ross, 2026). Healthcare students, educators, and providers express their desire and need for better integrated, more inclusive and accurate LGBTQIA+ content in healthcare curricula (Wynn *et al*., 2024; Priddle *et al*., 2025; Brown and Ross, 2026).

Despite the barriers, there are clinicians who work in safe and affirming ways with LGBTQIA+ communities. It is vital to learn from these clinicians and gain insight into how to improve practice. This study seeks to explore the key features of LGBTQIA+ affirming care from the perspective of primary care providers with demonstrated experience and expertise in LGBTQIA+ health.

## Methods

### Theoretical underpinnings

This qualitative, interpretative study was grounded in constructivist methodology, underpinned by queer theory and relativism. We used queer theory as a critical lens through which we could interrogate or challenge the norms, structures and language that shape healthcare for LGBTQIA+ people (e.g., binary, systems, power, and language). Using relativist epistemology, we acknowledged that all perspectives are individual and valid. This means we centred and prioritized the experiences and meaning-making of participants within their own professional, cultural and social contexts. This lens allowed for the exploration of complexity, nuance and contradiction across participant narratives.

### Participants

Primary care providers working in Australia who self-identified as having expertise and experience working with LGBTQIA+ communities were recruited online via social media through the research teams’ personal and professional networks. By primary care, we refer to the first services people seek healthcare from outside of hospital or specialist settings. Primary care includes the diagnosis and treatment of health conditions, long-term care, and health promotion and prevention services. We are purposefully adopting a broad definition of primary care to include general practitioners, pharmacists, community health workers, social workers, allied health professionals, and many more (Australian Government Department of Health, Disability, and Ageing, 2023). Of those expressing interest and providing contact details, participants were invited sequentially, with purposive sampling used to ensure representation across a diverse range of primary care professions and participant characteristics. Recruitment continued until sufficient depth and repetition in the data were achieved. Participants provided informed consent both online and verbally prior to the interview, and the study was approved by The University of Queensland Human Research Ethics Committee (approval number: HE000130).

### Data collection

Data were collected online via semi-structured interviews conducted using Zoom. The interview guide was initially developed by RV and MHR, then reviewed and revised by the broader project team and consumer investigators. The interview guide was designed to explore clinicians’ experiences, understandings and practices in relation to LGBTQIA+ affirming care provision (Supplemental File 1). Interviews were between 24- and 68-min duration, were audio and video recorded, and transcribed verbatim within the videoconferencing software. Transcripts were de-identified prior to analysis, with profession and self-reported gender identity and sexual orientation retained to contextualize quotes in terms of professional and personal lived-experience.

### Data analysis

We took a relativist approach to data analysis, treating the data as deeply contextual and representative of participants’ experiences whilst not seeking any essential truth or reality (Braun and Clarke, 2022). The analyses presented in this paper are one interpretation of the data of many. Member checking was undertaken to support credibility and ensure participants’ voices were represented accurately, by sharing transcripts with participants for review and editing. Any revisions were incorporated in the final transcripts prior to analysis.

Themes, patterns of meaning or shared ideas present in the dataset, were generated following the six phases of reflexive thematic analysis outlined by Braun and Clarke (2022). The analytic process was recursive, meaning there was not always a fixed point where one phase ended, and another phase started allowing us to move between phases during analysis (Braun and Clarke, 2022). Data were analysed inductively, allowing the content of the data to inform the coding and theme development.

In the first phase, data familiarization, two authors (AH and MHR) independently read through all transcripts, taking notes on anything of interest. A1 and A2 met and discussed preliminary notes and initial insights from the transcripts. AH then began the second phase (coding) and coded the transcripts in NVivo software using a mix of semantic and latent coding. Towards the end of the second round of coding, a brief descriptive summary of the preliminary codes was written and discussed among the broader research team prior to theme generation. In the third phase, A1 began generating initial themes through thematic mapping, which involved clustering and re-organizing the code labels to explore various connections and common ideas between them, incorporating early ideas from the descriptive summary to group code labels. During this phase, two emergent overarching concepts were identified, to which each code was mapped. In phase four, AH and MHR met to discuss and refine initial themes by checking the themes against the coded data to see if they were true to the dataset. In phase five, AH and MHR met regularly to refine, name themes, and integrate the overarching concepts into an iceberg model inspired by existing frameworks of organizational cultures (Braithwaite, 2011) before finalizing the results in phase six.

### Research team reflexivity

The research team includes health professionals, researchers and consumers. This study is part of a larger multiphase project where consumers are engaged not only as advisors but co-investigators. Several members of the research team are members of LGBTQIA+ communities. All team members are non-Indigenous to Australia. Our diverse professional and personal identities informed the research project, and we engaged in ongoing reflexive discussions to interrogate assumptions, power and privilege, ensuring alignment with the theoretical underpinnings.

The last author (MHR), who identifies as queer, with experience as a health professional and qualitative researcher in LGBTQIA+ health, conducted all interviews. This insider position facilitated rapport and shared understanding with participants, while also requiring critical reflection to minimize assumptions and overfamiliarity. The first author (AH) is a white queer trans person with prior critical health research experience, but no professional background in primary care. MHR and AH engaged in reflexive conversations to consider how our lived experience as queer people informed our interpretations and priorities during data analysis. Throughout the study, the team viewed knowledge as co-constructed, positioning participants as active contributors to meaning-making rather than passive subjects. Peer discussions across the team enabled critical examination of interpretations, acknowledging that our differences in lived-experience or disciplinary background shaped the analysis.

## Results

### Participants

Thirty-one providers between 24 and 69 years of age, primarily living and working in large cities (27/31, 87.1%) in Victoria and Queensland (12/31, 38.7% each) participated in the study. Participants had collective experience across 12 primary care professions and self-identified with a range of gender identities and sexual orientations. Aggregated participant demographics are provided in Table 1.

**Table 1.** Participant demographics (*n* = 31) Table 1 long description.

Demographic	*n* (%)
*Primary care profession*	
General practice	7 (22.6)
Psychology	5 (16.1)
Speech pathology	4 (12.9)
Social work	3 (9.7)
Physiotherapy	2 (6.5)
Dietetics	2 (6.5)
Medical imaging	2 (6.5)
Occupational therapy	2 (6.5)
Social work; Counselling	1 (3.2)
Pharmacy	1 (3.2)
Arts Therapy	1 (3.2)
Paramedicine/nursing	1 (3.2)
*Gender identity*	
Woman	18 (58.1)
Man	3 (9.7)
Nonbinary; genderqueer	3 (9.7)
Nonbinary	2 (6.5)
Nonbinary; genderqueer; agender	1 (3.2)
Genderfluid	1 (3.2)
Genderqueer	1 (3.2)
Nonbinary; genderfluid	1 (3.2)
Woman; genderfluid	1 (3.2)
*Sexual orientation*	
Straight	8 (25.8)
Queer	6 (19.4)
Pansexual	5 (16.1)
Lesbian	4 (12.9)
Bisexual	4 (12.9)
Gay	3 (9.7)
Asexual	1 (3.2)
*Ethnic ancestry, heritage or background*	
Australian	19 (61.3)
European	3 (9.7)
Australian, European	3 (9.7)
Asian, European	2 (6.5)
North America	1 (3.2)
Indian	1 (3.2)
African	1 (3.2)
Asian	1 (3.2)
*State*	
VIC	12 (38.7)
QLD	12 (38.7)
NSW	4 (12.9)
TAS	1 (3.2)
SA	1 (3.2)
WA	1 (3.2)
*Geographical location*	
Large city	27 (87.1)
Regional	4 (12.9)

### Main findings

**Figure 1.**
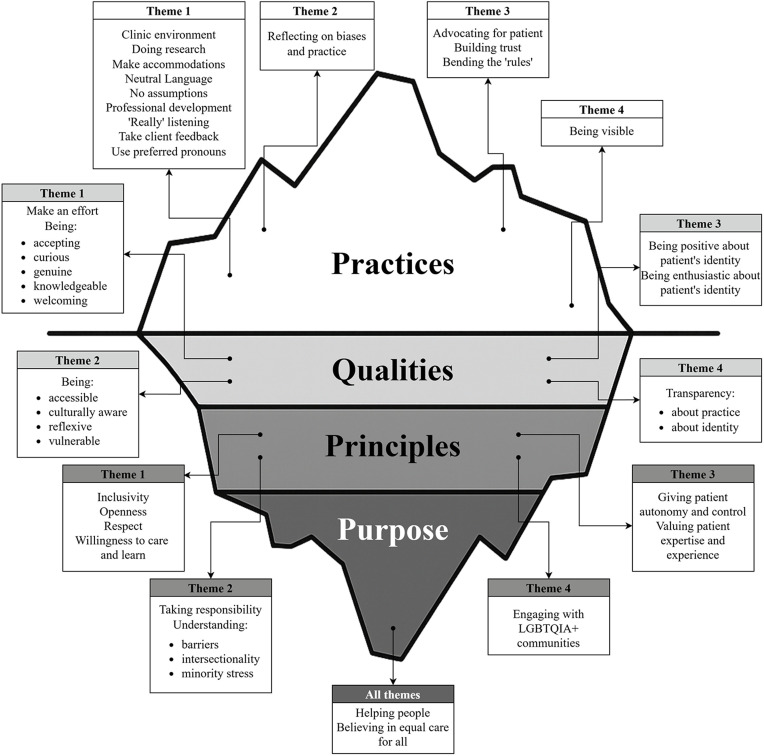
Iceberg model of affirming care, depicting the multiple levels of ‘practices’ and ‘values’, inspired by existing frameworks of organizational cultures (Braithwaite, 2011).

Two overarching concepts were generated from the analysis which were evident across all themes. These were named ‘practices’ and ‘values’. Many participants described straightforward, practical elements to how they made their care affirming. These actions were the tangible ways participants carried out affirming care. We refer to these as ‘practices’, which make up the ‘top’ of the iceberg and sit above water, because they are clearly visible and observable (Figure 1). Participants also discussed intangible elements of their care that informed their practice and were essential to being affirming and safe providers. We have named these ‘values’, that make up the underlying, unseen, below water portion of the iceberg because they underpin participants’ ‘practices’.

To capture the diversity in these values, we grouped them into specific categories: purpose, principles, and qualities. By purpose, we refer to descriptions of participants’ personal motivations that drive them to provide LGBTQIA+ affirming care or, broadly speaking, to work as healthcare providers. For example: *‘my main motivation… I think* ***I have always liked connecting with people*** *and* ***making their lives better***, *so I feel like pharmacy is one of the professions that we can do that.’* (Pharmacist, cisgender woman, heterosexual). Principles are deeply held foundational beliefs that informed how participants provide affirming care. For example, one participant said: *‘So we’ve all got that, as clinicians,* ***we all have that responsibility***, *and* ***we should all be stepping up*** *for all of those communities. Regardless of whether we’re a part of them or not.* (Speech pathologist, nonbinary/genderqueer, queer). By qualities, we refer to providers’ personality or personal characteristics that facilitated the provision of affirming care. For example: *‘just being really* ***curious*** *and* ***open*** *to, you know, that person’s experience, even that might be a bit different to your understanding.’* (Art therapist, nonbinary/genderqueer/agender, asexual).

How we have grouped the ‘values’ is not fixed. These groupings are shaped by the context in which the participants described why a particular value was important for their affirming practice. Furthermore, they have been grouped according to our interpretations and therefore the distinctions between purposes, principles, and qualities are only meaningful in the context of this study, as we explored the different ways participants’ values informed their affirming practice.

In the current study, themes give insight into the values participants described and how they practiced safe and affirming healthcare. Four themes were generated in relation to the key factors that underpin the provision of LGBTQIA+ affirming care that spanned both overarching concepts: practices and values.

**Theme 1:** Values-driven healthcare

Participants described the different ways that providing healthcare in line with their values and principles created a safe and affirming clinical space for LGBTQIA+ people. One example of many was using inclusive language:

*‘… being careful around how we word things in… antenatal education, and being conscious of blended families and rainbow families, [because] not everyone has… a standard male female relationship… So, being quite conscious in that language, so that it is inclusive in the way that we engage in the community. And… that filters through website, blog post, anything that we might publish. But then also the actual patient experience.’* (G.P., cisgender woman, heterosexual)

Here, the practical elements such as using inclusive or gender-neutral language to avoid making assumptions about patients’ families are how providers enact or embody inclusivity in practice. Other participants connected qualities like curiosity and openness to practices like asking patients open questions and active listening. They would contrast these practices against making assumptions or being rigid, especially in reference to patients’ gender and sexuality. One participant uniquely described their open and accepting approach to healthcare as ‘unflappable’:

*‘And I think this is hard to maybe put a finger on, but being just so unflappable about everything that comes up. […] I think that being casual about this kind of stuff is really important, because… we assume nothing. I mean… accepting… is the word. But that’s the word that I think also gets used a lot in this space, and can be hard to operationalize. […] I think that’s really what I’m meaning… just saying… yeah, okay? Oh, of course. And although that makes sense… not having an oh, that’s weird, or how strange kind of response, trying to be very… conversational, even faced, even humoured about what folks might be raising.’* (Psychologist, cisgender man, gay)

These intangible values such as being open or being welcoming are demonstrated here through the participants’ actions when they calmly and respectfully respond to patients, even when their first instinct might be to react with discomfort or surprise. Many participants described practices such as listening to patients, making accommodations, and adapting clinical forms, which were all underpinned by their desire to operationalize their values and principles of inclusivity, openness, and respect. These are ways providers can embody their values and show LGBTQIA+ patients that they want to provide safe and affirming care.

Participants acknowledged that no one is an instant expert in providing care, and that being willing to try and learn is important:

*‘Most trans and gender diverse and LGBT+ people will appreciate that you’re trying. That’s the main thing is that they just want to see people care, and that they try, and you can learn and progress with time.’* (Nurse/paramedic, genderfluid/trans, queer)

The participant explained that LGBTQIA+ patients understand that providers will make mistakes when a subject area is new to them, which implicitly alleviates the pressure providers might feel to immediately provide faultless care or use perfect language. This participant also emphasizes that following up ‘trying’ with action is crucial, because the process that accompanies values-driven healthcare is not passive, but active. Participants continually brought up the importance of following through on ‘trying’ by learning more about LGBTQIA+ health and upskill over time. Examples that participants gave for how other providers might improve were through professional development, doing research, and engaging with LGBTQIA+ communities. Relatedly, participants also often distinguished between ‘tokenistic’ gestures and genuine affirming care:

*‘I think it’s important to investigate the intention behind it, whether they’re actually wanting to be affirming because they care about improving health outcomes for queer people or it’s something that’s actually very meaningful to their… ethos and clinic views, and not just because they’re trying to tick a box of saying… Yeah, we technically will support queer people, but they’re not actually knowing what that entails or how they’re looking to reassess that and follow that up.’* (Physiotherapist, nonbinary, lesbian)

This participant critiques organizations that seek to support and affirm LGBTQIA+ people at a surface level to project a good image of their practice and ‘tick a box’. Many participants made similar critiques, often linking tokenism back to flawed intentions and expressing that good intent, to improve LGBTQIA+ health outcomes, was key to delivering good, values-driven healthcare. Participants contrasted their own willingness and motivation to provide safe and affirming care with others tokenistic approaches to caring for LGBTQIA+ patients. They again emphasized the active nature of developing affirming practice, that other providers and organizations could demonstrate their good intentions by taking action to improve their practice.

**Theme 2:** (Un)learning biases: Reflection and sociocultural awareness

A key concept that participants discussed was ‘bias’, and how crucial it was that health providers reflect on their unconscious biases to provide safe and affirming care. These discussions generally centred around two topics related to bias: 1) understanding how patients are impacted by stigma, bias and discrimination, and 2) reflecting on their biases and privileges in the healthcare space.

*‘acknowledging the social determinants of health and the way that, being aware of the health disparities based on things like minority stress and other overlapping minority groups that people might be a part of is going to be fairly important. Being aware of… the fact that our communities are at higher risk of suicide by… a really significant amount. Being aware of the impacts of relationships with family, safe and stable housing, financial security, all of that stuff.’* (Dietician, woman/nonbinary, bisexual)

Here, the participant mentions minority stress as one of the potential impacts of LGBTQIA+ marginalization that health providers should know about. Many participants stressed the importance of understanding how minority stress might play a role in LGBTQIA+ patients’ health. The participant also mentions that patients might experience multiple forms of ‘overlapping’ marginalization because of their identities. Other participants also valued intersectional approaches to care:

*‘[The] other thing that I think is really vital is understanding the intersectionality and the common overlaps in our community. [My] area of practice is neurodivergence and gender in particular, because we know there’s a very high overlap in those populations…[Also] with chronic illness and the nuances of working with First Nations people… I think you can’t… untangle those parts of our identity… And… if we’re talking about LGBTQ affirming practice, we can’t do it without talking about being safe and inclusive for other parts of people’s minority experiences.’* (Social worker/counsellor, nonbinary/genderqueer, queer)

This participant notes some common overlapping groups from their own practice (i.e., neurodiversity, chronical illness, and First Nations peoples). Participants identified a diverse range of marginalized realities that their LGBTQIA+ patients lived with, such as disability, ethnic and cultural diversity, rurality (or proximity to city centres), and more. Thus, many participants discussed how tackling the impact of stigma and discrimination in primary care requires an intersectional understanding of LGBTQIA+ health. Additionally, participants perceived it was important for providers to reflect on their biases towards LGBTQIA+ patients and how that bias might influence their practice:

*So I really think it takes… reading books, going to PDs, listening to queer people. Just yeah, really making our struggle, your struggle… And I just see so many people just putting flags on their website or saying they offer affirming support, but without doing the work.’* (Art therapist, nonbinary/genderqueer/agender, asexual)

Here, the participant expresses another issue with tokenistic approaches to LGBTQIA+ healthcare, whereby providers may naively believe they are delivering affirming care for LGBTQIA+ people but haven’t done the training or reflection required to do so. For many participants, taking responsibility for one’s learning as a provider and developing reflexivity informed the practical actions they took to reflect on their practice and their biases.

**Theme 3:** Mitigating the power imbalance in the patient–provider relationship

Providers’ reflections on their biases and practices prompted them to consider the role the medical system plays in perpetuating or facilitating discrimination towards LGBTQIA+ patients. Some participants discussed how part of developing reflexive practice involved understanding their position of privilege as a healthcare provider and how that impacts the relationship between themselves and their patients:

*‘understanding that there is that inherent power imbalance between a clinician and a patient and that very often, they’re already in a vulnerable state. They often bring vulnerable things. And so making that place safe is important for people to access care. But it’s also really important, clinically, because if you don’t build this relationship. You don’t get the information that you need to then provide good care for that person.’* (G.P., cisgender woman, heterosexual)

This participant expresses the additional care they take to create a safer place for patients in their practice, because of their position of privilege and power relative to their patients. They express the importance of the provider-patient relationship to good care, because building a sense of safety will encourage patients to share information with providers which facilitates better clinical decision-making and allows them to provide better care. Another important component of the patient–provider relationship providers identified was acknowledging and valuing patient’s experiences:

*‘I do think… there needs to be a change in the balance of power in healthcare, and I think… if you look at the very paternal relationships that doctors would have with patients, 40-50 years ago to now it is much more of a partnership. So, I think we need to continue in that sort of a direction where you’re having, yes, you’re bringing training and knowledge to the interactions that you have with clients and patients. But you do need to acknowledge their experience and not necessarily place the training and knowledge that you have above their needs and wishes.’* (Speech pathologist, nonbinary/genderqueer, queer)

Here, the participant highlights the principle of valuing patients’ expertise and lived experience, especially when making decisions about their care. Participants recognized and took responsibility over the privilege that is afforded to them as a provider. They expressed the value in acknowledging the power dynamic that is inherent to health practice, rather than obfuscating it. This understanding motivated some participants to advocate on their patient’s behalf, using their position to better support patients in difficult situations. For example:

*‘We will advocate. We will write letters, little mini passports of: Please be aware this is… Billy. Billy is trans man, currently pregnant blah blah blah … how to refer to them when you’re speaking with them. How to refer to them with your networks. They’re not a show pony. Please don’t bring every med student around… that sort of thing.’* (Psychologist, nonbinary/genderfluid, pansexual)

Here, the participant is describing how they use their privilege and authority as a health professional to help alleviate stress or difficulty their patients experience. Advocating for patients is an important element of primary care practice. This action protects patients from having to explain or defend themselves from other uninformed or ignorant providers. It also demonstrates some of the many ways participants described leveraging their relative power to actively empower patients who might not otherwise have spoken up when facing discrimination and stigma in healthcare spaces.

**Theme 4:** The value of lived experience

Not all participants were members of LGBTQIA+ communities. Those who were, however, often discussed the role of their lived experience in their practice. For many, drawing on their lived experience was vital to providing safe and affirming care:

*‘And then people around me, knowing about the community, about some of the challenges we face, or even just the basics of, if someone tells me about their gender or sexuality, I’m likely to understand what that means. I’m likely to use language that’s affirming to them, and or recognize when I need to be asking questions and helpful questions to ask… I think a safe space for LGBTQ + people is one where you’re not having to explain or justify your existence.’* (Dietician, woman/nonbinary, bisexual)

Here, the participant explains that their lived experience gives them a greater understanding of what language LGBTQIA+ people might use to describe themselves and what language is likely to be appropriate when talking to them. This knowledge helps them create a safer, more affirming environment in their practice for LGBTQIA+ patients. Several participants described the noticeable impact on their patients’ when they disclosed or signalled their LGBTQIA+ identity to patients:

*‘And I can feel that like you can really get that sense of… relief because and I know that because for some of my clients, they don’t know that I’m queer when they come in to see me, and then… during the session I will tell them, and you can just physically see this relaxing that happens. And… the armour has come down.’* (Social worker, woman, queer)

This participant highlights that their lived experience creates the opportunity to meaningfully connect with LGBTQIA+ patients through their shared experiences and, crucially, that this helps to build good rapport and trust with their patients. This connection was one of the benefits participants attributed to being visibly LGBTQIA+ in their workplace, whether that was through subtle signals (e.g., posters and flags in their office) or more overt signs (e.g., wearing pronoun pins or advertising one’s lived experience on practice websites).

Participants discussed that allies could also be visible in a different way, by being transparent about providing safe and affirming care to LGBTQIA+ people through similar means (e.g., wearing badges or displaying LGBTQIA+ health specific qualifications on one’s website). Visibility and transparency were intertwined as key values that informed many participants’ approaches to safe and affirming care.

For some participants, their lived experience of accessing healthcare as an LGBTQIA+ person was core to what inspired them to provide affirming care:

*‘It came out of both lived experience, wanting to be… a supportive practitioner, and someone who was out and safe, and… also from not seeing that many other queer practitioners in healthcare as I felt more comfortable when… I knew people were queer and could relate to me, and I kind of wanted to replicate that as well.’* (Physiotherapist, nonbinary, lesbian)

Here, the participant’s experiences informed their underlying purpose or reason for why they provide affirming care and, consequently, shaped their decision to be out as a queer practitioner. This sentiment, that lived experience was a driver of participant’s motivation to practice affirming and safe care was echoed by several LGBTQIA+ participants and thus influenced a great number of principles and qualities that underpinned affirming care. However, participants also emphasized that lived experience was not the single most important element of providing safe and affirming care. One participant talked about a colleague who was not a member of LGBTQIA+ communities but had lived experience of marginalization because of her neurodivergence:

*‘But… I think about… one of my staff members who is het [heterosexual], but neurodivergent and she does amazing work with trans people. […] the feedback we get is so beautiful. And about her creating safety, and my thoughts about that is that because she has experienced a world of ableism and discrimination around who she is that she has a really first hand lived experience of what that’s like to navigate, and so can really connect in that way. And I guess that’s… the reason we say the two communities is such a crossover and navigating those two identities in accessing services is so hard, but I think for us… just naturally, we would have someone who sits in one of those two communities because it impacts you as a person and when you’re interviewing and you’re working, you’re working then from a space of knowledge, of what oppression feels like.’* (Social worker, woman, queer)

Therefore, as captured in this quote, lived experience alone does not make for good healthcare, but it bridges a gap in knowledge between the provider and the patient. Providers without lived experience can be equally as capable at creating an affirming and safe space as those with it, even though they might take a different approach or draw on different experience. What participants described as essential to providing that care and creating that space is developing knowledge and expertise, not necessarily how those skills are acquired. Many LGBTQIA+ participants discussed the complexities that come with providing care for LGBTQIA+ patients. As one participant said:

*‘I’m more vulnerable to vicarious trauma because some of the things that my clients… will speak about, I would have directly experienced myself. [I]t’s really hard to not be affected by someone’s experience of discrimination and abuse because of their identity… [w]hen you’re part of that community.’* (Social worker, woman, queer)

For LGBTQIA+ providers, leveraging one’s own lived experience can be valuable for patients, but it may also create risk for the provider themselves. For example, the participant above describes one of many challenges and risks that LGBTQIA+ providers navigate when caring for members from their own communities. Other participants also discussed burnout.

Moreover, LGBTQIA+ participants acknowledged the limits of the knowledge obtained through lived experience, often explaining the positions of privilege they occupied. As one participant said:

*‘I’m a white middle class, highly educated man. I wield, despite being a member of the LGBTQ community within that community outside it, I still wield a great deal of power. One thing… I certainly go away and do my own… reflection, or seek out my own… training or reading. […] If we’re if we’re straying into an area that’s outside of my area of lived and perhaps even professional expertise, I’ll be asking, am I? Am I right? Am I wrong? Please correct me. What have I missed?’* (Psychologist, cisgender man, gay)

This participant – and several other LGBTQIA+ participants – were mindful of the intersectional experiences and insights that were missing from their personal perspective and were comfortable acknowledging those gaps in their expertise. Furthermore, some participants cautioned against assuming that lived experience is a substitute for taking steps to develop one’s skills and challenge one’s unconscious biases. For example:

*‘Because that it means that they, the person who has to say… is… queer themselves, is supporting queer people has done that work around like reflection, and their own internalized or externalized biases, stigma privileges. Yada, Yada, Yada. Which not all of them have. I know that because I talk to people who don’t. Despite the fact that they [are] queer or neurodivergent themselves, whatever respective community that they’re working with and they may be… reinforcing… cis het norms onto a person otherwise, because they haven’t done that.’* (Dietician, nonbinary/genderqueer, queer)

Here, the participant emphasized how important it is for all providers, regardless of lived experience, to work on unlearning their unconscious biases to prevent or mitigate the impact of unchecked biases on patients. Though lived experience was central in shaping how LGBTQIA+ providers engaged with their patients, the expertise they had was also from working on their practice and developing reflexivity.

The contribution of lived experience to making care safe and affirming is complex and messy. Participants who weren’t members of LGBTQIA+ communities often discussed how much they had learned from community members, whether from their own patients or from doing their own research in LGBTQIA+ people’s experiences. For example, one provider explained that listening to podcasts about LGBTQIA+ health topics from community members helped them embed safe and affirming care into their practice:

*‘I like to listen to a lot of podcasts and stuff… while I’m commuting so just hearing other people’s stories and other people’s point of view. […] It’s just really interesting to listen to the actual… real people, stories behind that. And how these… decisions and the research that set around that area, and what we know and don’t know… actually impacts real people.* (Medical imaging, cisgender woman, heterosexual)

For this participant, LGBTQIA+ people’s stories were a source of information on what affirming practice looked like. As this quote reflects, stories of LGBTQIA+ people’s lived experiences contextualized and made visible – or ‘real’ – the impact of affirming practice on LGBTQIA+ patients. Thus, each participant had a unique relationship with the concept of lived experience and how salient it was to making their practice safe and affirming.

## Discussion

This study used reflexive thematic analysis to explore the key features of safe and affirming care from the perspective of primary care providers who deliver such care to LGBTQIA+ people. Participants described the purposes, principles, and qualities (values) that informed and shaped how they provided affirming care (practices). These overarching tenets of affirming care were present in all the themes generated from our analysis. In undertaking this study, we expected that participants would describe practical aspects of providing affirming care, but did not anticipate the depth and consistency in descriptions of how their values informed their clinical practices. Our findings align with the broader literature which highlights the importance of provider attitudes to providing LGBTQIA+ affirming care, but expand on this by demonstrating *how* values and practices operate together as an integrated foundation for affirming care provision.

A key finding from this study was the relationship between participants’ values and their practices. We conceptualized this finding using the Braithwaite (2011) iceberg model of organizational cultures (see Figure 1), to show the interplay between practice, personal qualities, principles, and purpose (or broadly, values). The *National Action Plan for the Health and Wellbeing of LGBTIQA+ People 2025–2035* highlights the importance of building workforce capacity to provide safe and affirming care for LGBTQIA+ people, including through formal training, continuous professional development, and the integration of LGBTQIA+ health content into health professional education curricula in consultation with LGBTQIA+ communities and people with lived experience (Australian Government Department of Health, Disability and Ageing, 2024). However, our findings also suggest that education interventions might be more effective for changing practitioners’ behaviours if they were also connecting with providers’ values, as the iceberg model (Figure 1) suggests. This finding is congruent with research on affirming healthcare that suggests that education interventions that focus on imparting knowledge alone are insufficient for changing provider attitudes, behaviours and practices (Yu *et al*., 2023; Yu *et al*., 2023). Provider education should integrate behaviour change techniques to support affirming clinical practice by emphasizing cultural companionship, cultural humility, and general clinical capabilities rather than continuing to focus solely on highly specific or narrow LGBTQIA+ health content (Kuzma *et al*., 2019; Franks *et al*., 2023; Kimber *et al*., 2025).

Participants expressed that understanding the impact of social and political factors on health and wellbeing helped them provide better care. Several participants referred to frameworks such as minority stress and intersectionality to contextualize LGBTQIA+ people’s health disparities (Crenshaw, 1991; Meyer, 1995, 2003; Testa *et al*., 2015). LGBTQIA+ discrimination and marginalization not only create stressors that diminish wellbeing, but has shaped the health policy environment that historically neglected the health needs of LGBTQIA+ communities (Meyer, 2003; Ayhan *et al*., 2020). These factors have long shaped the quality of and access to healthcare for LGBTQIA+ people (Ayhan *et al*., 2020). By understanding these frameworks, providers can better reflect on the biases they may hold towards LGBTQIA+ people and better appreciate the ongoing impact of homophobia and transphobia on LGBTQIA+ communities.

Beyond understanding factors external to healthcare, participants emphasize the importance of reflecting on their healthcare practice. Health providers’ reflexivity about their practice and position in healthcare is crucial to affirming care (Kuzma *et al*., 2019). Reflecting on practice is important to ensure they are not inadvertently marginalizing LGBTQIA+ patients within the healthcare space itself. Curtis *et al*. (2019:14) posit that health practitioners and organizations can achieve health equity by striving for cultural safety, requiring providers and organizations to examine themselves, to ‘acknowledge and address their own biases, attitudes, assumptions, stereotypes, prejudices, structures and characteristics that may affect the quality of care provided.’ The authors go on to define cultural safety as a process of critical consciousness for health professionals and organizations, requiring ongoing self-reflection and self-awareness (Curtis *et al*., 2019:14). The minority stress model, intersectionality principles, and other frameworks for health equity (see, for instance, Quint *et al*., 2025) can be helpful for providers to understand how their practice could be improved in this regard. By engaging in reflexivity, providers can go beyond ensuring they aren’t perpetuating harm and instead aim to provide good, culturally safe, affirming care (Curtis *et al*., 2019; Kuzma *et al*., 2019).

Practitioners expressed that it was crucial for safe and affirming care that they were reflexive about their position of authority as healthcare providers. They acknowledged that the health provider position came with inherent power over patients, and that naming that power was one action they could take to facilitate a safer and more equal relationship with patients. One of the fundamental aspects of providing affirming care to LGBTQIA+ people for participants, and in broader literature, is providers building a good relationship with patients (Kuzma *et al*., 2019; Franks *et al*., 2023). By demonstrating humility and understanding the limits of their expertise, providers can build better relationships with patients (Huynh *et al*., 2021). Additionally, when providers give LGBTQIA+ patients autonomy over their care, they facilitate a more collaborative relationship between patient and provider, rather than undermining the patient and minimizing their lived experience (Kuzma *et al*., 2019; Quint *et al*., 2025).

Another finding from this study was that most participants valued LGBTQIA+ people’s lived experience with health systems, regardless of whether they identified as LGBTQIA+ themselves. Pearce (2018), citing Hess (2004) and Hanssmann (2016), argues that health professionals who are also members of the communities – insider-providers – are the drivers of change in the medical field because they can shape policies and protocols in medicine in line with community interests. Consumer consultation is an increasingly popular ‘indirect form’ of intervention (Pearce, 2018:163) to improve health services. Insider-providers in positions of leadership in healthcare organizations have the credibility to directly implement changes needed to facilitate safe and affirming practice, acting as a ‘direct form’ of intervention (Pearce, 2018:163). LGBTQIA+ providers in positions of epistemic authority can redirect resources and narratives within their organizations (Hess, 2004; Hanssmann, 2016). This relative ‘power’ is limited to how supportive and affirming providers’ immediate management, government, and socio-political climate is. However, providers can be involved in effecting and advocating for incremental systemic change in their professional capacities (Pearce, 2018). Thus, it is vital that LGBTQIA+ providers occupy leadership roles throughout every facet of primary care, from curriculum development at the university level through to sitting on association boards. Primary care organizations should value the work insider-providers do and support them to facilitate organizational change.

Many participants expressed that the support of colleagues, leaders, and institutions is vital to enable healthcare providers to enact their values into practice and deliver safe and affirming care. This support was particularly instrumental for LGBTQIA+ participants, who make up the majority of affirming providers despite the increased risk of vicarious trauma and burnout (Eliason *et al*., 2018). Participants with lived experience recommended strategies to mitigate these risks, such as clinical supervision and having safe people to debrief with at work. Organizations should implement additional supports for LGBTQIA+ providers working in affirming care to facilitate safer workplaces and prevent psychosocial harm to workers, particularly given the additional emotional labour and minority stress LGBTQIA+ professionals may experience within healthcare settings (Nicotera *et al*., 2020; Ross *et al*., 2022).

Our findings should be interpreted in context of where and when the data were collected, as LGBTQIA+ healthcare is a rapidly changing space in the current socio-political climate. A limitation of this research is the lack of ethnic diversity in our sample as this will have shaped our participants experiences of delivering care and interacting with the health systems, which in turn influenced our findings. Additionally, the findings will have been influenced by many of our participants being part of LGBTQIA+ communities which is not a limitation of the research itself, but further context for interpreting the findings. Our sample is made up of mostly women and nonbinary people which reflects what we know about who provides affirming care; that providers with existing ties to LGBTQIA+ communities tend to be more aware of their needs and invested in delivering affirming care. A strength of this study is the sizable sample of participants, as it can be difficult to recruit clinicians for research.

## Conclusion

Primary care providers participating in this study described both their affirming practices and their underlying values that constitute what safe and affirming care for LGBTQIA+ people was to them. From an organizational culture perspective, the iceberg model describes how values (purposes, principles, and qualities) shape behaviours and practices. Themes describe and explore key features (both unobservable and observable) that are important for safe and affirming care for LGBTQIA+ people. This research highlighted ways to improve healthcare provision by addressing both observable, and unobservable, factors which are vital to redress the health disparities caused by stigmatizing and discriminatory care. In doing so, this work provides critical insights into safe and affirming care for LGBTQIA+ people in primary care.

## Supporting information

10.1017/S1463423626101601.sm001 Hectors et al. supplementary material Hectors et al. supplementary material

## Supplementary material

The supplementary material for this article can be found at https://doi.org/10.1017/S1463423626101601.

## Table 1. Long description

A table summarizing participant demographics across various categories. The table has 31 rows and 2 columns. The columns are labeled ‘Demographic’ and ‘n (%)’. The table is divided into several sections based on different demographic categories: Primary care profession, Gender identity, Sexual orientation, Ethnic ancestry, heritage or background, State, and Geographical location. Each section lists specific categories and their corresponding values in terms of the number of participants (n) and the percentage (%). For example, under Primary care profession, General practice has 7 participants (22.6%), Psychology has 5 participants (16.1%), and so on. Under Gender identity, Woman has 18 participants (58.1%), Man has 3 participants (9.7%), and various nonbinary and genderqueer identities have smaller percentages. Sexual orientation includes categories like Straight, Queer, Pansexual, Lesbian, Bisexual, Gay, and Asexual with varying percentages. Ethnic ancestry includes Australian, European, and other backgrounds. States listed are VIC, QLD, NSW, TAS, SA, and WA with VIC and QLD having the highest number of participants. Geographical location is divided into Large city and Regional, with Large city having 27 participants (87.1%).

Navigate back to Table 1..
