# Supplementary material for: Aligning values and behaviours in LGBTQIA+ affirming care: primary care providers’ perspectives
Source: Prim Health Care Res Dev. 2026 Aug 7;27:e88. doi: 10.1017/S1463423626101601 (PMC13463159; doi:10.1017/S1463423626101601)
Supplement: Supplementary file 1 [file S1463423626101601sup001.docx]

**Supplemental File 1. Interview guide**

**Preamble:**

- Name, pronouns, background
- Interview will be about how they provide LGBTQIA+ affirming care
- There are no right or wrong answers, we are interested in hearing your perspectives
- If you want to take a break, or end the interview at anytime, you’re welcome to, just let me know
- So that we can analyse the data, are you happy for me to record the interview?

**Profession, motivations and values**

1. What is your profession? Your current role?
2. What motivated you to work in [this profession]?
3. What motivates you to work at your current workplace[/s]?

**Affirming Care provision**

4. What motivates you to practice affirming and safe care for LGBTQIA+ consumers/ clients / patients?
5. Can you describe what makes your own practice [or your organization] affirming and safe for LGBTQIA+ consumers/ clients / patients?
6. Can you describe what elements are essential to provide safe and affirming care for LGBTQIA+ consumers/ clients / patients? [prompts: the approach (e.g. person-centred; people; resources; built environment; services; etc.]
7. As a practice/organization, can you tell me how you embedded a culture of safe and affirming care in your workplace? [probes: hiring; interdisciplinary team; activities; training; maintenance; etc.]
a) What were the barriers, and how did you overcome them?
b) What were the facilitators, and how did you leverage them?
8. What are the key steps/processes you would recommend to another practice/organization who wanted to implement a safe and affirming model of care into their everyday practice?
a) What is important before? [prompts: readiness; values; upskilling; lived experience; etc.]
b) What is important during? [prompts: team building; feedback; etc.]
c) What is important after? [prompts: maintenance; evaluation, continuous improvement, etc.]

**Primary care readiness**

9. Thinking about primary care overall, do you think it is:
   a. well-trained to provide affirming care for LGBTQIA+ consumers/ clients / patients
   b. effective at providing affirming care for LGBTQIA+ consumers/ clients / patients
10. Is LGBTQIA+ affirming care a priority for primary care? [please elaborate/ tell me more]
    a. Where / how did you access / engage in LGBTQIA+ training? [please provide details]
11. From your perspective, is primary care safe for:
    c. LGBTQIA+ consumers/ clients / patients?
    d. LGBTQIA+ professionals?
12. What broader system changes do you think are necessary for primary care to embrace safe and affirming care provision?
    a. Medicare, PBS, professional bodies, Government, funding, etc
    b. How constrained are healthcare providers by these systemic factors in providing affirming care? How does this influence your ability to provide care?
    c. Who’s role is it (in your opinion) to affect systemic change?
